# Pharmacist’s interventions in factors contributing to medication errors reduces medication errors in self-management of patients in the rehabilitation ward

**DOI:** 10.1186/s40780-022-00268-5

**Published:** 2022-12-12

**Authors:** Ryohei Suzuki, Takako Uchiya, Takamasa Sakai, Masaaki Takahashi, Fumiko Ohtsu

**Affiliations:** 1 Department of Pharmacy, National Hospital Organization Higashinagoya National Hospital, 5-101 Umemorizaka, Meito-Ku, Nagoya, Aichi Japan; 2 Drug Informatics, Faculty of Pharmacy, Meijo University, 150 Yagotoyama, Tempaku-Ku, Nagoya, Aichi Japan

**Keywords:** Pharmacist intervention, Medication factor, Self-management, Medication error

## Abstract

**Background:**

The number of medications, number of administrations per day, dosing frequency on indicated day, and medication from multiple prescriptions are the medication factors prone to medication errors in self-management that have been previously reported. However, whether pharmacists actually intervene in medication factors that affect medication error occurrences in self-management is unclear. Therefore, we conducted this study to clarify these issues.

**Method:**

This study included patients who underwent self-management in the rehabilitation ward of Higashinagoya National Hospital. From April 2019 to March 2020, a one-pharmacist period existed, and from April 2020 to March 2021, a two-pharmacist period existed. The number of patient instructions and interventions were expected to increase with an increase in the number of pharmacists. Considering this to be an environment of differential interventions by pharmacists, a pre-post-test design was conducted with all self-managed patients in both the time periods. The primary and secondary endpoints were the proportion of medication error occurrences and proportion of pharmacist’s interventions in medication factors, respectively.

**Result:**

The proportions of medication error occurrences during the one-pharmacist and two-pharmacist periods were 41% (71/173) and 28% (51/180) (relative risk 0.690, 95% confidential interval 0.515–0.925), respectively. The proportion of pharmacist’s interventions in medication factors in the one-pharmacist period was 13% (22/173) and 22% (40/180) in the two-pharmacist period; there was an increase in the proportion of pharmacist’s interventions in medication factors in the two-pharmacist period.

**Conclusion:**

The proportion of medication error occurrences was significantly lower in the two-pharmacist period than that in the one-pharmacist period. This can be attributed to the increase in the proportion of pharmacist’s interventions in medication factors. Therefore, an environment in which pharmacists could intervene in the medication factors to prevent medication errors in advance is necessary.

## Background

The number of patients with chronic diseases and the need for continuous medication is increasing with age. Self-management of medication (self-management) is required because of the increase in the number of elderly people living alone or in households with only elderly couples, due to the shift to nuclear families. Additionally, self-management is conducted due to the patients’ desire to face their own disease and manage their own medications. Self-management has been beneficial in improving patient satisfaction, medication adherence, and self-care skills [1]. Furthermore, medication errors are found to be lower when patients self-manage medication than when administered by nurses [2]. Therefore, considering patients’ life after discharge, self-management training is provided from the time of admission. However, there is a report that a patient forgot or mistakenly took medication during self-management. [1].

A relationship between medication factors such as related medication has been reported [3, 4]. In addition, the number of medications and administrations per day, Medication Regimen Complexity Index (MRCI), which indicates the complexity of prescribing in self-management were reported to be associated with adherence and medication error [5–9]. The MRCI is complex to score, and its items do not include medication and prescribing factors, such as medication from multiple prescription orders and one-packaging, which are problems in clinical practice. Therefore, we conducted a case–control study to identify the factors contributing to medication errors based on the hypothesis that these medication error factors, which are a problem in daily clinical practice, might contribute to medication errors, using patients who made a medication error as the case group and patients who did not make medication errors as the control group [10]. Consequently, it was previously found that medication factors, specifically, the number of medications, number of administrations per day, dosing frequency on indicated days (on alternate days or less frequently), and medication from multiple prescriptions (orders prescribed by more than one physician and/or by the same physician at different times) were associated with medication errors, and that the traditionally mentioned factors of patient cognitive and motor functions were less related to medication errors [10].

On the other hand, there are reports that pharmacist confirmation of prescriptions contributed to the reduction of medication errors when prescribing [11], pharmacist medication counseling improved medication adherence, [12, 13] and pharmacist’s interventions to review prescribing content reduced the MRCI score [14].

However, it is unclear whether pharmacists actually intervene in medication factors that affect the proportion of medication error occurrences in self-management. This study aims to address these issues.

## Methods

### Research design

This was a pre-post test design.

### Subject of the survey

To prospectively examine this study, we need to compare the proportion of medication errors between instances in which pharmacists intervened in medication factors and instances in which pharmacists did not intervene. However, it is not ethically feasible for pharmacists to avoid intervention.

In the rehabilitation ward of Higashinagoya National Hospital (60 beds), there was one pharmacist A with 3 years of experience from April 2019 to March 2020 and there were two pharmacists, pharmacist A with 4 years of experience and pharmacist B with 7 years of experience from April 2020 to March 2021. Starting in April 2020, the two pharmacists divided the beds into two equal groups and regularly checked the medication therapy of all inpatients and evaluated them in terms of therapeutic effect, adverse effects, interactions, and renal and hepatic function, with reference to various guidelines. The pharmacist checked the pharmacotherapy, suggested prescriptions to the physician, and made changes to the prescription based on the patient's and family's wishes and medication status if intervention was required. The increase in the number of pharmacists had naturally led to an increase in the number of patient instructions and associated interventions in prescriptions. Then, all patients who self-managed medications in the two time periods of April 2019 to March 2020 with one ward pharmacist and April 2020 to March 2021 with two ward pharmacists were included to compare whether differences in the intensity of the pharmacist’s interventions had an effect on the proportion of medication error occurrences during self-management. In this study, self-management was defined as self-management of more than one day’s worth of medication.

### Methods of medication self-management

In the rehabilitation ward of Higashinagoya National Hospital, physicians, pharmacists, and nurses checked the type and number of medications that patients were administered, their Functional Independence Measure (FIM) [15], and a Mini-Mental State Examination (MMSE) [16] from the perspective of medical safety. Self-management training was conducted when healthcare professionals determined that the patients were capable of taking medication. When starting self-management, pharmacists and nurses provided information such as the name of the medication, its purpose, number of administrations, timing, and adverse effects. They also trained patients on self-management by ensuring that they could open their medications on their own and understand when to take them appropriately. The criteria for initiation of self-management did not change during the study period.

### Patient background and environment of healthcare professionals

This study surveyed the following patient backgrounds from electronic health records: age, sex, primary medical department, methods of medication management on admission, length of self-management of medication, length of hospital stay, mini-mental state examination (MMSE), functional independence measure motor (FIM-M) score, which is the sum of 13 items related to exercise, and functional independence measure cognition (FIM-C) score, which is the sum of 5 items related to cognition.

This study also surveyed the environmental factors affecting healthcare professionals as the number of physicians, nurse staffing structure, number of medication counseling and instructions by pharmacists per month in the rehabilitation ward during the study period.

### Primary outcome and method of measurement

The primary endpoint was the proportion of medication errors occurring in patients undergoing self-management. The method to check for medication errors was to instruct the patients who were self-managing to not discard the packages of the medications after taking them. Nurses confirmed a medication error by subsequently checking the packages at each dosage time to ensure that correct medications and doses were taken at the correct time, as prescribed during the self-management training. Additionally, when medication errors occurred, the nurse recorded the details of the errors. Therefore, we extracted the cases of medication errors during self-management by reviewing all nursing records in the electronic health records of patients who were self-managed during the study period. This study then determined and compared the proportion of medication error occurrences during one-pharmacist and two-pharmacist periods. The proportions of medication error occurrences were calculated with the denominator as the number of self-managed patients during the study period and the numerator as the number of patients who had at least one medication error. Based on the American Society of Health-System Pharmacists classification system, medication errors during self-management were defined as omitted medication, taking the incorrect medication, taking it at an incorrect time, and/or taking an incorrect dose [17].

### Secondary outcome and the method of measurement

The secondary endpoints were the proportions at which pharmacists’s intervened prior to medication errors in patients’ medication factors and the details of the intervention. Pharmacist’s interventions after medication errors were excluded from the study.

### Proportion of pharmacist’s interventions in medication factors

This study surveyed pharmacist’s interventions in medication factors from the pharmacist record. Intervention in the medication factors was defined as one or more of the following items: the pharmacist made a prescription suggestion to the physician that resulted in a reduction in the number of medications or administrations per day, resolved the dosing frequency on indicated days, and resolved the medication from multiple prescriptions. The resolution of dosing frequency on indicated days specifically means reviewing the usage every other day or less frequently. The resolution of medication from multiple prescriptions specifically means that when there are multiple prescriptions, the dispensing process should be the same as when a single prescription is dispensed, with one-packaging and re-dispensing. This study then compared the proportions of pharmacist’s interventions in the one-pharmacist and two-pharmacist periods. The proportion of pharmacist’s interventions in the medication factors was calculated with the denominator as the number of self-management patients during the study period and the numerator as the number of patients who intervened in at least one of the four medication factors. We focused on pharmacist’s interventions in the medication factor; thus, patients who received some other intervention or instruction based on pharmacological knowledge were counted as patients for whom the pharmacist did not intervene in the medication factors.

### Details of pharmacist’s intervention in the medication factors

In the one-pharmacist and two-pharmacist periods, the patients were divided into ones with and without medication error. Furthermore, they were also divided based on whether there was an intervention by the pharmacist on the medication factor, including the details of the intervention.

### Statistical methods

In this study, the patient backgrounds such as the age, length of hospital stay, length of self-management of medication, MMSE, and FIM score in the one-pharmacist period and two-pharmacist periods were compared using the Mann–Whitney U test. Sex and primary medical departments, methods of medication management on admission were compared using the χ2 test. The χ2 test was used to compare the proportion of medication error occurrences for self-management. The significance level was set at 5%. All data were analyzed using IBM SPSS Statistics version 27 (IBM Corporation).

### Ethical considerations

This study was approved by the Ethical Review Committee of the National Hospital Organization Higashinagoya National Hospital.

## Results

### Patient background and environment of healthcare professionals

During the one-pharmacist period, there were 461 inpatients, of whom 173 were self-managed. During the two-pharmacist period, there were 438 inpatients, of whom 180 were self-managed.

The backgrounds of the self-managed patients in each period are shown in Table 1. A comparison of patient backgrounds for each period showed no differences in age, sex, methods of medication management on admission, length of self-management of medication, FIM-M, and FIM-C at admission and discharge. However, there were differences in the number of patients admitted to the neurosurgery department and MMSE. The number of physicians during the one-pharmacist and two-pharmacist periods were 8 and 10, respectively. The nursing staff of the wards remained constant in both periods, with one nursing staff assigned to every 13 inpatients. The number of medication counseling and instructions per month during the one-pharmacist and two-pharmacist periods were 89 and 231, respectively. During the two-pharmacist period, there were 117 and 114 medication counseling and instructions per month for pharmacists A and B, respectively.

**Table 1:** Patient background and environment of healthcare professionals

		One-pharmacist period		Two-pharmacist period		*P*
Patient background		*n* = 173		*n* = 180		
Age, median (IQR), years		75	(66–82)	74	(64–82)	0.733 ^a)^
Sex, n (%)	Men	87	(50)	90	(50)	0.957^b)^
	Women	86	(50)	90	(50)	
Length of hospital stay, median (IQR), day		44	(29–64)	42	(29–63)	0.998 ^a)^
Methods of medication management on admission						
Self-management from admission, n(%)		39	(23)	51	(28)	0.212 ^b)^
Nurse-management from admission, n(%)		134	(77)	129	(72)	
Length of self-management of medication, median (IQR), day		27	(16–42)	28	(17–42)	0.558 ^a)^
Primary medical department, n (%)						
Neurology		75	(43)	90	(50)	0.211 ^b)^
Orthopedic Surgery		67	(39)	73	(41)	0.726 ^b)^
Neurosurgery		31	(18)	17	(9)	0.020 ^b)^
MMSE, median, (IQR), score		27	(25–29)	28	(26–30)	0.041 ^a)^
Unknown, n (%)		27	(16)	23	(13)	
FIM, median, (IQR), score						
FIM-M	Admission	61	(51–71)	61	(52–75)	0.383 ^a)^
	Discharge	83	(77–87)	84	(79–88)	0.383 ^a)^
FIM-C	Admission	31	(28–34)	32	(29–35)	0.101^a)^
	Discharge	34	(31–35)	34	(32–35)	0.120 ^a)^
Environment of healthcare professionals						
Number of physicians, n		8		10		
Nurse staffing structure (Patients: Nurse)		13:1		13:1		
Number of medication counseling and instructions per month, n		89		231		

IQR, interquartile range; MMSE, Mini-Mental State Examination; FIM-M, Functional Independence Measure-Motor; FIM-C, Functional Independence Measure-Cognition; a) Mann–Whitney U test; b) χ^2^ test

### Primary outcome

**Fig. 1:**
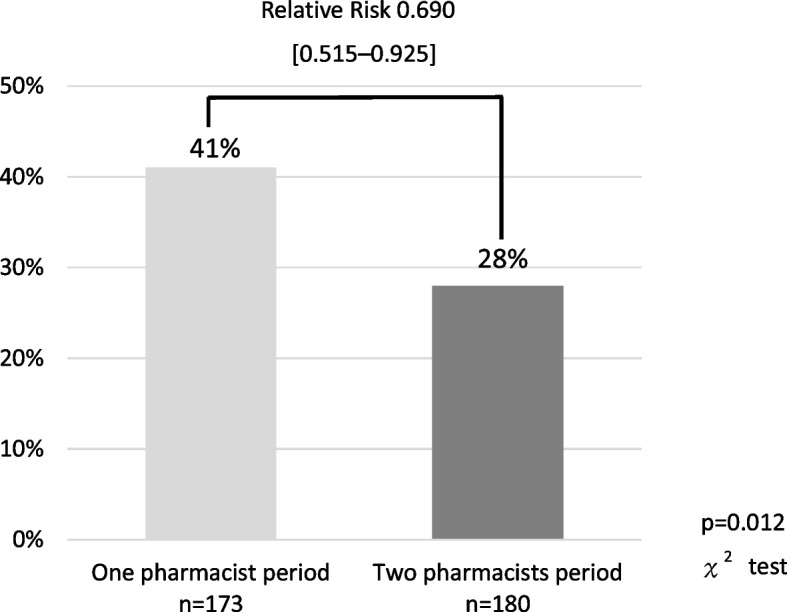
Proportion of medication error occurrences

The proportions of medication error occurrences during the one-pharmacist period and two-pharmacist period were 41% (71/173) and 28% (51/180), respectively (relative risk 0.690, 95% confidential interval [CI] 0.515–0.925, *P* = 0.012) (Fig. 1).

### Secondly outcome

The overall proportions of pharmacist’s interventions in the medication factors in the one-pharmacist period and two-pharmacist period were 13% (22/173) and 22% (40/180), respectively. In addition, pharmacists A and B intervened in 19 and 21 patient cases, respectively. In the one-pharmacist period, nine patients (5%) had a medication error despite the pharmacist’s interventions, and 13 patients (8%) did not have a medication error. In the two-pharmacist period, 11 patients (6%) had a medication error despite the pharmacist’s interventions, and 29 patients (16%) did not have a medication error, indicating a substantial increase. Contrastingly, in the one-pharmacist period, 62 patients (36%) had medication errors with no pharmacist’s interventions, and 89 patients (51%) did not have medication errors with no pharmacist’s interventions. In contrast, during the two-pharmacist period, 40 (22%) patients had a medication errors, which was a significant decrease from the one-pharmacist period due to an increase in the proportion of pharmacist’s interventions in the medication factors (Table 2).

**Table 2:** Proportion of pharmacist’s interventions in medication factors

	One-pharmacist period *n* = 173						Two-pharmacist period *n* = 180					
	Total		Patient who had a medication error		Patient who did not have a medication error		Total		Patient who had a medication error		Patient who did not have a medication error	
Patients for whom the pharmacist intervened prior to medication error in the patient's medication factor^*^,n (%)	22	(13%)	9	(5%)	13	(8%)	40	(22%)	11	(6%)	29	(16%)
Patients for whom the pharmacist did not intervene in the patient's medication factors, n (%)	151	(87%)	62	(36%)	89	(51%)	140	(78%)	40	(22%)	100	(56%)

^*^Patients for whom the pharmacist intervened prior to medication error in the patient's medication factor: Patients for whom the pharmacist intervened in at least one of the four medication factors

### Pharmacist’s interventions details for medication factors

Table 3 shows the breakdown of the pharmacist’s interventions in the medication factors in the one-pharmacist and two-pharmacist periods, divided into patients who had and did not have a medication error. In particular, when pharmacists intervened to reduce the number of administrations per day, there were no medication errors in 19 of 23 patients in the two-pharmacist period compared to 3 of 6 patients in the one-pharmacist period. In addition, in the case where medication from multiple prescriptions was resolved, there was no intervention in the one-pharmacist period, but there were interventions in three patients and no medication errors in two patients in the two-pharmacist period.

**Table 3:** Details of pharmacist’s intervention in the medication factors

	One-pharmacist period *n* = 173			Two-pharmacist period *n* = 180		
	Total	Patient who had a medication error	Patient who did not have a medication error	Total	Patient who had a medication error	Patient who did not have a medication error
Patients who were intervened to reduce the number of medications, n	21	8	13	28	8	20
Patients who were intervened to reduce the number of administrations per day, n	6	3	3	23	4	19
Patients who were intervened to resolve the dosing frequency on indicated days, n	0	0	0	0	0	0
Patients who were intervened to resolve the medication from multiple prescriptions, n	0	0	0	3	1	2

## Discussion

The purpose of this study was to determine whether the intervention of the pharmacists in medication factors affect the proportion of medication error occurrences in patients undergoing self-management. There were no differences in the backgrounds of the self-managed patients in each period in terms of age, sex, methods of medication management, and length of self-management of medication. On the other hand, there was a statistical difference in MMSE scores, with 27 and 28 points in each period. However, the cutoff score for dementia in the MMSE was set at 23 [18], and the median MMSE score for patients in each period exceeded the cutoff score. MMSE scores and FIM-C correlate with cognitive function [19], and since FIM-C showed no significant difference, the influence of clinical cognitive differences between the two groups is considered to be small. The number of physicians in charge increased during the two-pharmacist periods, while the number of neurosurgery patients was lower because there was a period when neurosurgery physicians were temporarily unavailable and did not accept patients, and a neurology physician temporarily served as the attending physician. In addition, nurse staffing structure in the wards has not changed; thus, the environment of these healthcare professionals is not expected to have an impact on the occurrences of medication errors. The number of medication counseling and instructions by pharmacists was about three times greater in the two-pharmacist period than in the one-pharmacist period. The number of medication counseling and instructions by each pharmacist during the two-pharmacist period was similar, and there was little difference in ward duties between each pharmacist. Thus, it can be concluded that patient background and environment of healthcare professionals were the same in these two periods and that there was a quantitative difference in the intervention of pharmacists.

The primary endpoint of the proportion of medication error occurrences decreased significantly from 41% (71/173) in the one-pharmacist period to 28% (40/180) in the two-pharmacist period. The relative risk was 0.690, and the relative risk reduction was 0.310—that is, the risk of occurrences of a medication error was reduced by 31%. This can be attributed to the increase from 13% (22/173) to 22% (40/180) in the proportion of pharmacist’s interventions in medication factors in the two-pharmacist period compared to the one-pharmacist period. Particularly, compared to the one-pharmacist period, there was an increase in the proportion of patients who did not have a medication error with pharmacist’s interventions to reduce the number of administrations per day in the two-pharmacist period. It is believed that the two pharmacists in charge were able to evaluate the pharmacotherapy of all inpatients. Subsequently, to enable self-administration, the pharmacist combined the medications with different dose timings to the same time. There were also interventions, such as varying the amount of medication to be taken at one time so that it can be taken in one dose. This may have reduced the chances of erroneously taking the medication. A previous study reported that using an administration timing simplification protocol aimed at maintaining medication adherence in patients with chronic disease reduced the number of administrations and improved medication adherence [20]. Therefore, pharmacist’s interventions to reduce the number of administrations are considered effective in reducing medication errors.

In the two-pharmacist period, the number of patients who did not have medication errors increased from 13 (8%) to 29 (16%) due to the increase in the proportion of pharmacist interventions. This suggests that if the pharmacist determined that there was a medication factor that required intervention during pharmacotherapy, they intervened in the medication factor without any leakage, thereby preventing medication errors. The number of patients who had a medication error without pharmacist’s interventions decreased from 62 (36%) to 40 (22%). The reason for no intervention for these 40 patients was that they were taking therapeutically necessary medications for their primary disease or complication and could not reduce the number of medications, number of administrations per day, and dosing frequency on the indicated day, such as bisphosphonates once a week, and they had medication factors that could not be intervened upon. In these 40 patients, for whom intervention was not provided, it was important to improve medication support to avoid medication errors, maintain good adherence, and provide personalized medication management for each patient.

Nine patients (5%) in the one-pharmacist period and 11 patients (6%) in the two-pharmacists period had a medication error despite the pharmacist interventions. These patients were also taking therapeutically necessary medications for their primary disease or complications, and the inability to reduce the number of medications or the number of administrations and dosing frequency on the indicated day, such as bisphosphonates once a week, was often the cause of medication errors. Nonadherence has been reported to be caused by barriers such as patients’ beliefs and perceptions about their medications and disease [21] in addition to medication factors. If these factors are considered, assistance by pharmacists to remove these barriers is necessary.

This study has several limitations because of its retrospective design. We cannot exclude the influence of unknown confounding factors, except for those investigated in this study. Therefore, factors other than pharmacist’s interventions may reduce the occurrences of medication errors. In this study, the number of medication counseling and instructions increased because the number of pharmacists had increased, and there was an improvement in the patients’ own medication knowledge and disease awareness, which may also have contributed to the decrease in proportion of medication error occurrences. However, there was no survey regarding the influence of changes in patients' medication knowledge or disease awareness. The number and content of interventions may vary depending on the years of experience and skills of pharmacists, and there is a need for a large-scale study to be conducted at many facilities.

This study is considered to be the first report of a significant reduction in the proportion of medication error occurrences in patients undergoing self-management, as there was an increase in the proportion of pharmacist’s interventions in medication factors. The most effective way to prevent or reduce medication errors in hospital wards is to assign pharmacists to wards [22]. Thus, it is necessary to provide an adequate pharmacist so that pharmacists can determine if intervention is needed in medication factors for all inpatients. It is believed that pharmacist’s interventions in medication factors will help prevent the occurrences of medication errors.

## Conclusions

Pharmacists regularly need to evaluate the medication regimens of all inpatients, and there needs to be an increase in the proportion of interventions in medication factors that require intervention. This would ultimately reduce medication errors in self-management.

## Data Availability

All data generated or analyzed in this study are included in the manuscript.

## List of abbreviations

FIM: Functional independence measure
FIM-M: Functional independence measure-motor
FIM-C: Functional independence measure cognition
MMSE: Mini-mental state examination
